# Anti-Inflammatory and Antioxidant Effects of the Indole-Derived N-Salicyloyltryptamine on Peritonitis and Joint Disability Induced by Carrageenan in Rodents

**DOI:** 10.1155/2022/5524107

**Published:** 2022-05-13

**Authors:** B. P. Sousa-Neto, F. V. M. Cunha, Daniel Barbosa Nunes, B. S. Gomes, Layane Valeria Amorim, Everton Moraes Lopes, S. J. C. Gutierrez, F. R. C. Almeida, D. D. R. Arcanjo, M. F. V. Souza, F. A. Oliveira

**Affiliations:** ^1^Medicinal Plants Research Center, Federal University of Piaui, Av. Universitária s/n, Campus Ministro Petrônio Portella, SG-15, Ininga, 64049-550 Teresina, PI, Brazil; ^2^Faculty of Pharmacy, Federal University of Piauí, Av. Universitária s/n, Campus Ministro Petrônio Portella, Ininga, 64049-550 Teresina, PI, Brazil; ^3^Department of Biochemistry and Pharmacology, Federal University of Piauí, Av. Universitário s/n, Campus Ministro Petrônio Portella, SG-08, Ininga, 64049-550 Teresina, PI, Brazil; ^4^Department of Biophysics and Physiology, Federal University of Piauí, Av. Universitária s/n, Campus Ministro Petrônio Portella, Ininga, 64049-550 Teresina, PI, Brazil; ^5^Federal University of Paraíba, Coordinator of the Graduate Program in Development and Technological Innovation of Drugs. (Association UFRN/UFPB/UFRPE/UFC), 64049-550 Teresina, PI, Brazil

## Abstract

**Purpose:**

To investigate the anti-inflammatory and antioxidant activities of N-salicyloyltryptamine (NST) in experimental models of carrageenan (Cg)-induced peritonitis in mice, and evaluation of the effects of NST on Cg-induced joint disability in rats.

**Methods:**

Female Swiss mice were submitted to Cg-induced peritonitis in mice or Cg-induced joint disability in rats after intraperitoneal injection of NST (100 or 200 mg/kg). Total leukocyte count, total protein concentration, myeloperoxidase (MPO) and catalase (CAT) activities, and nitrite (NO_2_^−^) and thiobarbituric acid reactive species (TBARS) levels were determined.

**Results:**

NST significantly decrease the migration of leukocytes to peritoneal exudate. Cg induces inflammatory responses mediated by expression of reactive oxygen species (ROS). The results further showed that NST significantly decreased MPO and CAT activities, as well as reduced NO_2_^−^ and TBARS levels, compared with the vehicle group. Animals treated with NST significantly reduced paw elevation time (PET) on the first hour after induction of joint injury, and this effect was sustained throughout the analysis.

**Conclusion:**

NST presented anti-inflammatory and antioxidant effects in experimental models of carrageenan-induced peritonitis and joint disability in mice and rats, respectively, which may be related to the modulation of neutrophils migration as well as the involvement of antioxidant mechanisms.

## 1. Introduction

The indole nucleus is part of many natural and synthetic molecules with important biological activities [1]. Among the indolic derivatives, benzoyltryptamine, whose structures are related to N-benzoyltryptamine, is previously obtained from the species *Myrtopsis myrtoidea* (Baill.) Guillaumin, a plant of the family Rutaceae [2]. These derivatives include the compounds N-N-dimethyltryptamine and 5-methoxy-NN-dimethyltryptamine, which possess psychotomimetic activity [3]. N-Salicyloyltryptamine or (2-hydroxy-N-[2-(1H-indol-3-yl)ethyl]-benzamide) exhibited anticonvulsant, hypnotic, and muscle relaxant activities [4].

An in vitro study has shown that NST negatively modulates expression of both TNF-*α* and IL-1*β*, inhibits the phosphorylation of ERK1/2 and I*κ*B*α*, and reduces the concentrations of reactive oxygen (ROS) and nitrogen (RNS) species in macrophages, thus contributing to the reversal of inflammatory response [5]. The presence of oxidizing agents has been associated with cardiovascular diseases [6], diabetes [7], cancer, [8], rheumatoid arthritis, and arthrosis [9]. The study shows that NST exerts antiedematogenic activity in in vivo models induced by different phlogistic agents [10]. However, no studies have been found in the literature showing the antioxidant activity of NST in vivo protocols.

One of the main oxidizing agents present in the inflammatory process is nitric oxide (NO) [11], produced mainly by activated macrophages; when reacted with oxygen, it forms peroxynitrite (ONOO^−^), a powerful protein oxidant. ONOO^−^ can then be protonated in the presence of ionic hydrogen (H^+^), giving rise to a highly reactive and toxic radical, hydroxyl (HO^−^), effectively potentiating the toxic action of NO and O^−2^ [12]. This radical favor vasodilation due to a synergistic effect with PGI_2_, relaxing like muscle cells, allows the movement of leukocyte cells through the endothelium [13]. However, ONOO^−^ also produces harmful effects on the organism, such as tissue damage, DNA deamination, and activating nuclear signaling pathways for the transcription of proinflammatory agents [14, 15].

The most reasonable alternative to inhibit ROS is supplementation with antioxidant agents. However, it has often not been possible to attenuate the progress of the disease, reducing the life expectancy [16]. Oxidative disorders produced by ROS or RNS are underlying several inflammation-related pathophysiological processes by the production of proinflammatory cytokines and suppression of the antioxidant defense systems and characteristic edema formation [17]. In diseases such as rheumatoid arthritis and arthrosis, which normally occur in the joints, free radicals are produced primarily by macrophages and activated neutrophils in the synovial membrane and chondrocytes [9]. In this sense, there is a need for discoveries of new compounds with both anti-inflammatory and antioxidant potentials.

Therefore, considering that reports of the anti-inflammatory and antioxidant effects of NST in vivo assays involving the inhibition of reactive oxygen species are lacking, the present study aims to investigate the anti-inflammatory and antioxidant effects induced by NST in experimental models of peritonitis and articular disability in experimental models in rodents.

## 2. Materials and Methods

### 2.1. Drugs and Reagents

The NST 2-hydroxy-N-[2-(1H-indol-3-yl)-ethyl]-benzamide was obtained by “in house” synthesis as previously reported (João Pessoa, PB, Brazil; patent document: BR 200304393-A) in Laboratory of Pharmaceutical Chemistry, UFPI (Teresina, PI, Brazil) [5]. Carrageenan, acetic acid, Triton X-100, L-methionine, hydroxylamine chloride, riboflavin, thiobarbituric acid (TBA), Griess reagent, sodium dodecylsulfate, O-dianisidine, hexadecyltrimethylammonium bromide (HTAB), sodium phosphate, 5,5′-dithiobis-(2-nitrobenzoic acid) (DTNB), and sodium nitrite were purchased from Sigma-Aldrich (Saint Louis, MO, USA). Indomethacin (Indocid®), sodium phosphate, zinc sulfate, hydrogen peroxide, Tris base, sodium hydroxide, and trichloroacetic acid were purchased from Merck (Darmstadt, Germany). Turk's solution was purchased from Newprov (Pinhais, PR, Brazil).

### 2.2. Animals

Female Wistar rats (170–230 g) and Swiss mice (27–32 g) (*n* = 6 per group) were used in experiments. The animals were maintained at 23 ± 1°C and 12–12 h dark/light cycles, and they had free access to food and tap water. After experimental protocols, the animals were euthanized by intraperitoneal administration of thiopental sodium (150 mg/kg, i.p.). The conduction of all experimental protocols using animals was authorized by the Animal Use Ethics Committee (CEUA) from Universidade Federal do Piauí (permission no. 082/14; date of approval: 24-Nov-2014), as well as they followed internationally recommended guidelines.

### 2.3. Effect of NST on Carrageenan-Induced Peritonitis in Mice

Female Swiss mice (25–30 g) were treated intraperitoneally with vehicle (0.9% NaCl + 5.0% Tween 80), NST at doses of 50, 100, and 200 mg/kg), or indomethacin at dose of 10 mg/kg. After 30 minutes, carrageenan (0.05 mL at 1.0%) was administered into the peritoneal cavity [18]. Then, after 4 hours, euthanasias were carried out, and then, peritoneal cavities were washed with 5 mL of heparinized (10 IU/mL) PBS solution [19]. Additional exudate aliquot was collected to analyze the activities of myeloperoxidase (MPO), catalase (CAT), nitrite (NO_2_^−^), and thiobarbituric acid reactive species (TBARS).

### 2.4. Total Leukocyte Counting in the Peritoneal Exudate

A sample of 0.380 mL of the Turk's solution (1/20) was added to 0.02 ml of peritoneal exudate, and the number of leukocytes was counted using a Neubauer chamber under the optical microscope. Numbers of total leukocytes were determined according to the following formula: cells/mm^3^ = *N* × 20 × 2.5, where *N* represents the number of cells, 20 is the dilution factor of Turk's solution, and 2.5 is the total volume into the Neubauer chamber. Results were expressed as total leukocytes/mm^3^ [20].

### 2.5. Measurement of Proteins in the Peritoneal Exudate

Proteins were quantified using specific protein dosing kits [21]. Biuret reagent, potassium hydroxide solution (KOH), and copper sulfate (CuSO_4_), associated with sodium and potassium tartrate (KNaC_4_H_4_O_6_·4H_2_O), were used to dose proteins. After centrifugation of the exudate, the supernatants were collected and transferred to specific cuvettes and placed in a Labtest®'s automatic biochemical analyzer, serial number 1208.26 (Lagoa Santa, MG, Brazil), according to manufacturer's recommendations.

### 2.6. Evaluation of Myeloperoxidase Activity (MPO) in the Peritoneal Exudate

The MPO activity was assayed according with the method [21]. Briefly, 400 *μ*L of the peritonitis exudate was centrifuged at 4000 g for 7 min at 4°C. Then, 100 *μ*l of supernatant was collected and added to 1 ml of 0.5% HTAB buffer, pH 6.0 (hexadecyltrimethylammonium bromide), and centrifuged at 4500 ×*g* and 4°C for 10 min. Then, 10 *μ*l of the supernatant was withdrawn and incubated with 200 *μ*l of the reading solution (0.167 mg/mL O-dianisidine and 1.0% H_2_O_2_ in phosphate buffer pH 6.0). After 5 min, the MPO activity was determined at 450 nm and expressed in units of MPO per microliter (U MPO/*μ*L).

### 2.7. Evaluation of Catalase Activity (CAT) in the Peritoneal Exudate

Briefly, samples (20 *μ*L) were incubated with 50 mM phosphate buffer, pH 7.0 (1.2 mL). Right after, 1.0 mL of 30 mM H_2_O_2_ was added, and absorbances were measured at 240 nm every 2 min for 6 min. CAT activity was expressed in *μ*mols of H_2_O_2_ decomposed/min/*μ*L of peritoneal exudate [22].

### 2.8. Determination of Nitrite (NO_2_^−^) Content in the Peritoneal Exudate

Briefly, 20 *μ*L of the peritoneal exudate was added in distilled water (1 : 4 v/v) and submitted to deproteinization by incubation of 300 g/L zinc sulfate (1 : 20 v/v), followed by centrifugation for 15 min at 1000 ×*g* [23]. Afterwards, 100 *μ*L of Griess' reagent was added to 100 *μ*L of supernatant. After 10 minutes, absorbances were read at 550 nm [24]. A standard curve was obtained for NaNO_2_ solution (sodium nitrite) to determine nitrite concentration. Results are expressed in terms of *μ*mol/mL of peritoneal exudate.

### 2.9. Determination of Thiobarbituric Acid Reactive Species (TBARS) Content in the Peritoneal Exudate

For thiobarbituric acid reactive species (TBARS) determination, twenty microliters of the exudate were added to 0.5% TBA (600 *μ*L) and 20% acetic acid pH 3.5 (350 *μ*L). Then, samples were heated at 100°C and boiled for 45 minutes, followed by ice bath for 15 minutes. Afterwards, fifty microliters of 8.1% sodium dodecyl sulfate (SDS) were added, and samples were centrifuged at 12,000 ×*g* for 15 minutes at 25°C. Absorbances were read at 420, 490, and 550 nm filters. A standard curve was obtained for malondialdehyde (MDA) to determine the TBARS content. Results were expressed as nmol MDA/mL of peritoneal exudate [25].

### 2.10. Assessment of NST-Induced Effects on Carrageenan-Induced Joint Disability in Rats

In parallel with the carrageenan-induced peritonitis, the effects of NST on joint motor disability degree were evaluated. After setting for 30 minutes and immediately prior to the induction of carrageenan joint disability, the animals were submitted to the joint disability test for 60 seconds at the speed of 3 revolutions per minute (RPM), and the results were recorded as a control measure [26]. The animals were then treated intraperitoneally with vehicle (0.9% NaCl + 5.0% Tween80), NST at doses of 50, 100, and 200 mg/kg, or indomethacin at dose of 10 mg/kg. After 30 minutes, the animals received intraarticular administration of 100 *μ*L (300 *μ*g) of carrageenan into tibiofemoral joint of right paws. Measurements were performed for 60 seconds every hour until the sixth hour and one reading after 24 hours.

### 2.11. Statistical Analysis

The results were expressed as mean ± SEM. One-way ANOVA followed by Tukey's post-test or two-way ANOVA followed by Bonferroni's post-test were applied. Differences were considered at significance level when *p* < 0.05. Statistical analyses and graphs plotting were performed using GraphPad Prism® software version 6.01 for Windows (GraphPad Software, La Jolla California USA, https://www.graphpad.com).

## 3. Results

### 3.1. Effect of NST on the Total Number of Leukocytes in Carrageenan-Induced Peritonitis Exudate in Mice

**Figure 1:**
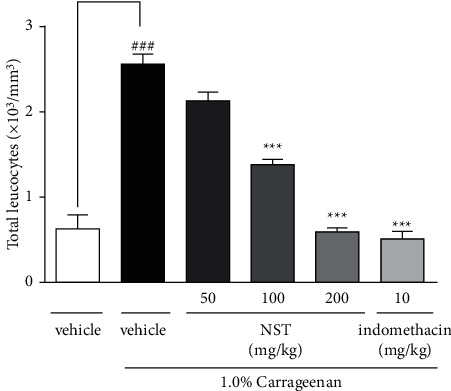
Effects of NST (50, 100, or 200 mg/kg, i.p.), vehicle (0.9% NaCl + 5% Tween 80, i.p.), and indomethacin (10 mg/kg, i.p.) on the total number of leukocytes in the peritoneal exudate of mice after administration of 1.0% carrageenan. Values are expressed as mean ± S.E.M. ^###^*P* < 0.001 when compared with only vehicle; ^*∗∗∗*^*p* < 0.001 when compared with vehicle plus 1.0% carrageenan. One-way ANOVA was followed by Tukey's post hoc test.

Pretreatment of animals with NST (100 and 200 mg/kg) was able to significantly reduce the migration of total leukocytes (1.37 ± 0.07 and 0.59 ± 0.05, respectively) to the peritoneal exudate, as well as for indomethacin (10 mg/kg) (0.5 ± 0.1), when compared with the vehicle group (2.56 ± 0.11). NST at the dose of 50 mg/kg was not effective in this protocol (Figure 1).

### 3.2. Measurement of Total Proteins in the Peritoneal Exudate

**Figure 2:**
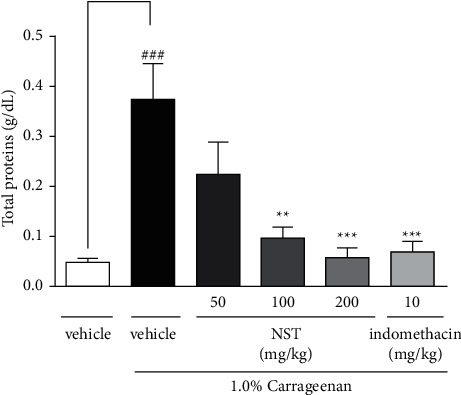
Effects of NST (50, 100, or 200 mg/kg, i.p.), vehicle (0.9% NaCl + 5% Tween 80, i.p.), and indomethacin (10 mg/kg, i.p.) on the concentration of total proteins in the peritoneal exudate of mice after administration of 1.0% carrageenan. Values are expressed as mean ± S.E.M. ^###^*P* < 0.001 when compared with only vehicle; ^*∗∗*^*p* < 0.01 and ^*∗∗∗*^*p* < 0.001 when compared with vehicle plus 1.0% carrageenan. One-way ANOVA was followed by Tukey's post hoc test.

Both NST at doses of 100 and 200 mg/kg or indomethacin (10 mg/kg) markedly reduced the concentration of total proteins in the peritoneal exudate when compared with the vehicle group. NST at 50 mg/kg was not effective in this protocol (Figure 2).

### 3.3. Effect of NST on Myeloperoxidase (MPO) Activity

**Figure 3:**
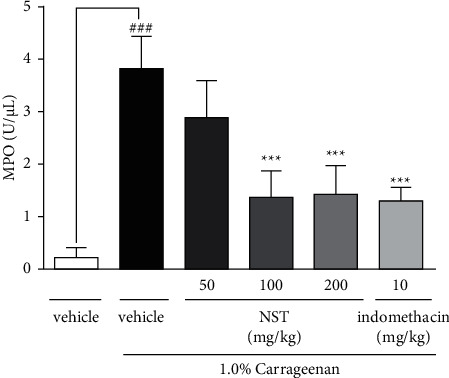
Effects of NST (50, 100, or 200 mg/kg, i.p.), vehicle (0.9% NaCl + 5% Tween 80, i.p.), and indomethacin (10 mg/kg, i.p.) on the myeloperoxidase (MPO) activity in the peritoneal exudate of mice after administration of 1.0% carrageenan. Values are expressed as mean ± S.E.M. ^###^*P* < 0.001 when compared with only vehicle; ^*∗∗∗*^*p* < 0.001 when compared with vehicle plus 1.0% carrageenan. One-way ANOVA was followed by Tukey's post hoc test.

Myeloperoxidase activity decreased in animals treated with NST (1.38 ± 0.20 and 1.43 ± 0.24 at doses of 100 and 200 mg/kg, respectively) or indomethacin (1.09 ± 0.20) when compared with the vehicle group (3.90 ± 0.31). No significant results were observed in the group treated with NST 50 mg/kg (Figure 3).

### 3.4. Effect of NST on Catalase (CAT) Activity

**Figure 4:**
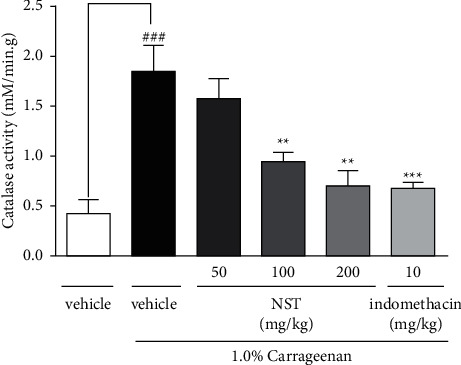
Effects of NST (50, 100, or 200 mg/kg, i.p.), vehicle (0.9% NaCl + 5% Tween 80, i.p.), and indomethacin (10 mg/kg, i.p.) on the catalase (CAT) activity in the peritoneal exudate of mice after administration of 1.0% carrageenan. Values are expressed as mean ± S.E.M. ^###^*P* < 0.001 when compared with only vehicle; ^*∗∗*^*p* < 0.01 and ^*∗∗∗*^*p* < 0.001 when compared with vehicle plus 1.0% carrageenan. One-way ANOVA was followed by Tukey's post hoc test.

In NST-treated groups (100 and 200 mg/kg), CAT activity significantly decreased (0.93 ± 0.10 and 0.71 ± 0.15, respectively) when compared with the vehicle group (1.84 ± 0.27). Treatment with indomethacin (10 mg/kg) significantly decreased catalase activity (0.69 ± 0.04) when compared with the vehicle group (1.84 ± 0.27) (Figure 4).

### 3.5. Effect of NST on Nitrite (NO_2_^−^) Concentration

**Figure 5:**
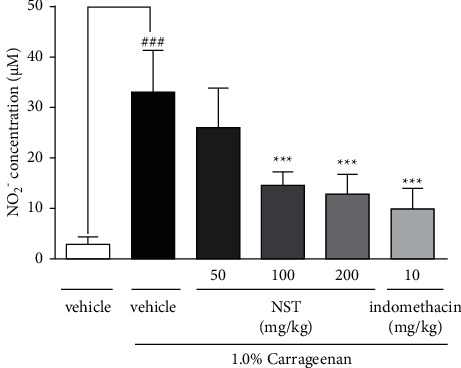
Effects of NST (50, 100, or 200 mg/kg, i.p.), vehicle (0.9% NaCl + 5% Tween 80, i.p.), and indomethacin (10 mg/kg, i.p.) on nitrite (NO_2_^−^) concentration in the peritoneal exudate of mice after administration of 1.0% carrageenan. Values are expressed as mean ± S.E.M. ^###^*P* < 0.001 when compared with only vehicle; ^*∗∗∗*^*p* < 0.001 when compared with vehicle plus 1.0% carrageenan. One-way ANOVA was followed by Tukey's post hoc test.

The NST at doses of 100 and 200 mg/kg promoted a significant decrease in the concentration of nitrite (14.09 ± 1.17 and 13.02 ± 1.51, respectively) in peritoneal exudate when compared with the vehicle group (33.05 ± 3.57). Likewise, animals treated with indomethacin (10 mg/kg) also showed a significant reduction of NO_2_^−^ concentration. On the other hand, NST 50 mg/kg did not present any significant effect (Figure 5).

### 3.6. Effect of NST on the Concentration of TBARS

**Figure 6:**
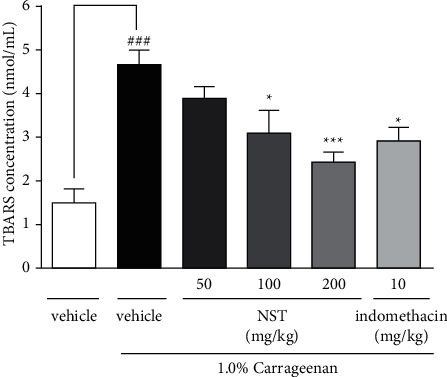
Effects of NST (50, 100, or 200 mg/kg, i.p.), vehicle (0.9% NaCl + 5% Tween 80, i.p.), and indomethacin (10 mg/kg, i.p.) on TBARS concentration in the peritoneal exudate of mice after administration of 1.0% carrageenan. Values are expressed as mean ± S.E.M. ^###^*P* < 0.001 when compared with only vehicle; ^*∗*^*p* < 0.05 and ^*∗∗*^*p* < 0.01 when compared with vehicle plus 1.0% carrageenan. One-way ANOVA was followed by Tukey's post hoc test.

NST (100 and 200 mg/kg) was able to decrease concentrations of thiobarbituric acid reactive species (3.10 ± 0.53 and 2.40 ± 0.24, respectively) when compared with the vehicle group (4.68 ± 0.32). Likewise, indomethacin (10 mg/kg) decreased TBARS concentration (2.94 ± 0.32). On the other hand, any significant results were observed for NST at the lowest dose of 50 mg/kg (Figure 6).

### 3.7. Effect of NST on Carrageenan-Induced Joint Disability

**Figure 7:**
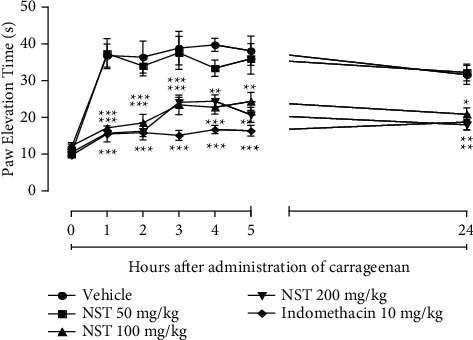
Effect of NST (50, 100 or 200 mg/kg, i.p.), vehicle (0.9% NaCl + 5% Twee n80, i.p.), and indomethacin (10 mg/kg, i.p.) on the carrageenan-induced joint disability 100 *μ*L (300 *μ*g) in female rats. Values are expressed as mean ± S.E.M. ^*∗∗*^*p* < 0.01 and ^*∗∗∗*^*p* < 0.001 vs. vehicle control. One-way ANOVA was followed by Bonferroni's post hoc test.

Injection of carrageenan (300 *μ*g per 100 *μ*L of total volume) into the tibiofemoral joint of the animals caused articular disability evidenced by an increase in paw elevation time in the vehicle group. In the present study, NST (100 and 200 mg/kg) and indomethacin (10 mg/kg) showed a marked significant decrease in paw elevation time (PET) during 4 h of observation, which remained up to the 24th hour after carrageenan injection. NST (50 mg/kg) showed no significant inhibition during the observed period (Figure 7).

## 4. Discussion

Compounds that contain the indole nucleus are related to the metabolism of tryptophan and may exhibit ligands in different regions of the indolic core [27]. The indole ring has a heterocyclic structure described in 1866, which is present in a considerable number of natural compounds derived mainly from plants [28]. NST consists of an N-benzoyltryptamine-related chemical structure, an isolated alkaloid from the plant species *Myrtopsis myrtoidea* (Baill) Guillaumin [2]. Furthermore, a recent study demonstrated no sign of apparent toxicity and no animal deaths after intraperitoneal administration of NST at 2000 mg/kg followed by 14 days of observation [10].

Indolic derivative compounds present in biological tissue such as tryptophan, melatonin, and serotonin are potent hydroxyl radical sequesters, preventing cellular oxidative damage and mitigating leukocyte migration [27]. Therefore, the carrageenan-induced acute peritonitis in mice was chosen for investigation of mechanisms underlying both antioxidant and anti-inflammatory effects of NST. When administered intraperitoneally, carrageenan was able to increase capillary permeability, which causes leukocyte infiltration, extravasation of proteins into the tissue, or exudate and release of cytokines. These events are important in triggering inflammatory processes [29]. In this context, this experimental model allows to quantify migrated cells towards peritoneal cavity, which involves roles of inflammatory cytokines and chemokines [30].

Considering the cellular events, the evaluation of the presence of leukocytes is important to assess the installation of an inflammatory response. Neutrophils play important and crucial roles in several inflammatory conditions [31]. In this study, animals pretreated with NST showed a marked reduction in both number of leukocytes and total proteins in peritoneal exudate. This may contribute to the recovery from the inflammatory process.

Then, the NST modulating activity on important enzymatic complexes such as myeloperoxidase (MPO), catalase (CAT), and nitrite (NO) concentrations and thiobarbituric acid reactive species were analyzed. MPO can be released after activation of leukocytes in phagosomes or in the extracellular space. When released, it reacts with the hydrogen peroxide formed by NADPH oxidase and increases the toxic potential of this oxidant [32]. A previous study has shown that substances that inhibit MPO activity may exhibit important anti-inflammatory activity [33]. In this study, similar results were observed in groups treated with NST or indomethacin, where MPO concentration was decreased, suggesting an anti-inflammatory activity of NST in this process.

The activity of the catalase in the peritoneal exudate was then evaluated. This enzyme catalyzes the reduction of H_2_O_2_ into H_2_O eliminating ROS as such this contributes efficiently to combat inflammatory processes [34]. The decrease in catalase activity was observed in the study. This may be related to the decrease of leukocyte cells in the peritoneal exudate, since part of this antioxidant enzyme is produced by mitochondria, mainly leukocytes [35]. This study shows that catalase activity is reduced in patients with decreased myeloid lineage cell; however, patients with leukocytosis from chronic myeloid leukemia had high catalase activity [36].

Later, the NST activity on nitrite concentrations in the peritoneal exudate was analyzed. NO-2 is a relatively short-lived reactive species which underlies several biological processes. As nitric oxide (NO), it has role as an important endothelium-derived relaxing factor and is responsible for regulating vascular blood flow promoting vasodilation [37]. Considering the involvement of NO in inflammation-related cellular and molecular events, the decrease of biosynthesis of this radical using NO synthase inhibitors, such as L-NAME, has been investigated as a potential treatment of inflammatory conditions [38]. Therefore, we investigated the action of NST on the concentration of nitrite in carrageenan-induced peritonitis in mice. Factors such as low concentration and extreme short half-life hinder the dosing of NO in biological samples [39]. Thus, the indirect determination can be made by plasma nitrate and nitrite dosage [40]. Animals intraperitoneally treated with NST promoted a significant reduction of nitrite concentrations at doses of 100 and 200 mg/kg. Interestingly, extraction fractions obtained from *Alstonia scholaris* L. containing alkaloids such as picrinine, valesamine, and escolaricin, which contain the indolic nucleus in their structures, also displayed the anti-inflammatory effect by inhibition of NO and PGE_2_ biosyntheses [41].

The antioxidant activity of NST on lipid peroxidation was also investigated; it is known that this phenomenon is quite harmful to the organism because it favors free radical reaction increasing cellular damage [42]. Scientific literature reports that the lipids peroxidation is directly associated with the onset of diseases such as atherosclerosis, rheumatoid arthritis, and Alzheimer's disease [43, 44]. The assessment of damages caused by lipid peroxidation on oxidative stress and inflammation was performed by determining thiobarbituric acid reactive species (TBARS) in experimental groups. In this sense, animals treated with NST showed significant lower concentrations of TBARS at doses of 100 or 200 mg/kg when compared with the vehicle group. Studies show that indole alkaloids such as 16-formyl-alpha-methoxystrictamine tubotaivine and picralinal inhibited exudation in xylene-induced mice ear edema. These alkaloids inhibited the concentrations of inflammation-related mediators, such as TBARS, PGE_2_, NO_2_^−^, and malondialdehyde in the experimental model of carrageenan-induced air pouch in mice [45].

Previous studies show that the presence of the oxidizing agent in the articular synovial fluid favors lipid peroxidation and induces chondrocyte degradation and cartilage peroxidation. These events represent a primary risk factor for the development of arthritis [46]. In parallel, neutrophil depletion in uric acid crystal-induced gout is important for reversal of the inflammatory process [47]. Thus, considering the involvement of the migration of neutrophils and decrease of oxidative stress, the effects of NST on carrageenan-induced joint disability in mice were evaluated. This test is based on the joint disability using the loss of functionality through the inflammatory process as a measure (functio laesa). This technique is widely used for screening of possible analgesic and anti-inflammatory drugs [48–50].

The induction of arthritis by intraarticular injection of carrageenan presents a pattern of development similar to carrageenan-induced mouse paw edema. The administration of carrageenan in the joint cavity promotes in the first hours the liberation of inflammation-related mediators which induce edema and fast infiltration of polymorphonuclear granulocytes, leading to disablement due to joint sensitization manifested by limb withdrawal when the inflamed joint is pressed (mechanical hyperalgesia) [51]. In this model of carrageenan-induced joint disability, NST was effective in reducing the paw elevation time (PET). NST-treated animals showed a decrease in joint disability from the first hour after administration of carrageenan.

Chemical compounds containing indole nucleus presented antinematode and anti-inflammatory activities by acting as inhibitors of 5-lipoxygenase and cyclooxygenase enzymes, which convert arachidonic acid to leukotrienes and prostaglandins, respectively [52]. Studies have reported that indole alkaloids have exhibited a wide range of biological activities, such as anti-inflammatory activity, because they inhibit superoxide anion generation and the release of human neutrophil elastase [50–53]. They also possess anticonvulsive, cardiovascular, and antibacterial properties [45]. Thus, the present study corroborates the previous findings, since we found that NST decreases the paw elevation time when compared with the vehicle group.

## 5. Conclusion

In this regard, the present study supports evidences of the anti-inflammatory action of NST and its possible benefits in treatment of joint disability. Furthermore, nonclinical and clinical studies may be relevant regarding its potential application as an effective anti-inflammatory drug and to ensure its safety for therapeutic purposes.

## Data Availability

The data used to support the findings of this study are available from the corresponding author upon request.
